# Integrative Analysis of Major Depressive Disorder and Ovarian Cancer: From Genetic Association to Single-Cell Mechanisms

**DOI:** 10.3390/biomedicines14051167

**Published:** 2026-05-21

**Authors:** Chen Liu, Xueling Wang, Jiaqi Lu

**Affiliations:** Department of Obstetrics and Gynecology, The Obstetrics & Gynecology Hospital of Fudan University (Shanghai Red House Ob & Gyn Hospital), Shanghai 200082, China; liu_chen_@fudan.edu.cn (C.L.); melodyofyou@163.com (X.W.)

**Keywords:** major depressive disorder, ovarian cancer, CLSTN3, mendelian randomization, single-cell RNA sequencing

## Abstract

**Background:** Although emerging evidence indicates that major depressive disorder (MDD) raises the risk of developing ovarian cancer (OC) and worsens survival, the biological mechanisms underlying this relationship remain unclear. This study explores the MDD-OC association using single-cell transcriptomics and genetic approaches. **Methods:** Using single-cell RNA-seq profiles of peripheral blood from MDD and OC patients, we compared shifts in immune cell subsets and selected the consistently expanded CD8^+^ effector memory (CD8_EM) T cells population for follow-up, validated using flow cytometry. We integrated expression quantitative trait loci (eQTL) data from CD8_EM T cell-specific genes with OC genome-wide association study (GWAS) summary statistics through two-sample Mendelian randomization (MR). In vitro experiments were additionally conducted to assess CLSTN3’s role in OC cell proliferation. **Results:** Among the 554 differentially expressed genes (DEGs) identified in CD8_EM T cells, MR showed a nominal association between CLSTN3 and ovarian cancer risk (OR 1.21, 95% CI 1.03–1.43), though this did not withstand correction for multiple comparisons. Colocalization analysis confirmed that CLSTN3 expression, regulated by the genetic variant rs3759416, shares a causal variant with the OC GWAS signal (PPH4 = 99.99%). Functionally, siRNA-mediated CLSTN3 silencing in HOC7 cells significantly reduced cell viability (CCK-8 assay). **Conclusions:** By focusing on CD8_EM T cells shared by MDD and ovarian cancer, we identified CLSTN3 as a candidate molecule through nominated by the convergence of genetic, transcriptomic, and functional evidence. These findings provide preliminary insights into the connection between depression and OC, though further validation is warranted.

## 1. Introduction

Ovarian cancer continues to rank among the deadliest gynecological malignancies, with its prevalence steadily increasing annually [1]. Beyond the well-established risk factors of family history and genetic predisposition, emerging evidence suggests that psychological distress, particularly major depressive disorder (MDD), may contribute to disease susceptibility and progression [2]. Accumulating research has demonstrated that MDD can disrupt neuroendocrine pathways, promoting ovarian tumor progression and metastasis through stress-activated molecular networks [3,4].

MDD is now recognized as a systemic condition that predisposes individuals to multiple somatic diseases, including cardiovascular disease [5], diabetes [6], arthritis [7] and, increasingly, cancer [8,9]. Through neuroendocrine dysregulation, immune surveillance impairment, and promotion of high-risk behaviors, depression intersects with fundamental processes in cancer development [10]. Depression is also linked to increased cancer risk in men, including associations with prostate, colorectal, and lung cancer [11]. These parallel findings suggest that the MDD–cancer relationship reflects shared biological vulnerabilities—particularly HPA axis hyperactivation and chronic inflammation—that may differentially influence tumor microenvironments across tissue types [12]. However, the molecular mechanisms linking MDD to specific cancer types remain poorly understood.

The relationship between MDD and ovarian cancer is clinically significant. Both conditions predominantly affect women, and epidemiological studies indicate a bidirectional association: MDD increases ovarian cancer susceptibility, while cancer diagnosis exacerbates depressive symptoms [13,14]. A meta-analysis of 8315 patients reported pooled prevalence rates of 35% for depression and 37% for anxiety in ovarian cancer populations [15], and comorbid depression has been associated with a 94% increased mortality risk [16]. Biologically, ovarian cancer exhibits distinct tumor microenvironment characteristics—including peritoneal dissemination, ascites formation, and a pronounced inflammatory milieu [17]—that are highly susceptible to modulation by chronic stress and depression-related pathways such as HPA axis hyperactivation and elevated systemic pro-inflammatory cytokines [12,18]. These features make ovarian cancer a particularly relevant model for investigating the MDD–cancer relationship.

Immune system alterations—particularly disturbances in T cell populations—appear to mediate this connection. Depression promotes the expansion of activated cytotoxic (CD3^+^CD8^+^CD69^+^) and exhausted (CD3^+^Lag3^+^) T cells while reducing naive and memory B cells, creating an immunological profile associated with unfavorable ovarian cancer prognosis [19,20,21]. Concurrently, tumor-induced pro-inflammatory cytokines (e.g., IL-6, TNF-α) drive both cancer progression and neuroinflammation, contributing to HPA axis dysregulation and depressive symptomatology [18].

Previous investigations into the genetic architecture connecting these conditions have been limited by bulk tissue analysis constraints. Recent advances in single-cell RNA sequencing (scRNA-seq) and genome-wide association studies (GWASs) now enable cell type-specific transcriptomic profiling and causal inference through Mendelian randomization (MR) [22]. This study integrates these complementary approaches to characterize the relationship between MDD and ovarian cancer relationship. Using UK Biobank data, we first established that depression is a significant risk factor for ovarian cancer, exhibiting a clear dose–response relationship. We then identify CD8^+^ effector memory (CD8_EM) T cells as a shared expanded population in both conditions through scRNA-seq, nominate CLSTN3 as a candidate gene via MR and colocalization analyses, and functionally validate its role in ovarian cancer cell proliferation through in vitro experiments.

## 2. Methods

### 2.1. Single-Cell Sequencing Data Processing

Peripheral blood mononuclear cell (PBMC) single-cell RNA sequencing datasets were acquired from two distinct sources: GSE264489 [23] provided samples from ovarian cancer patients and non-cancer controls, while PRJCA032578 [24] contributed data from major depressive disorder (MDD) patients and healthy individuals. The final cohort comprised three treatment-naïve ovarian cancer cases, six non-cancer controls, eight MDD patients, and eight healthy controls.

All computational analyses were conducted using R Studio (v4.4.1) with the Seurat toolkit (v5.3.1). Initial quality control procedures involved filtering cells based on established parameters: only cells containing between 200 and 2500 detected RNA molecules were retained to eliminate low-quality samples, and cells exhibiting mitochondrial gene content exceeding 5% of total transcript counts were systematically excluded. Following these stringent quality assessment steps, high-quality cellular profiles were preserved for subsequent analytical procedures.

### 2.2. Integration, Dimensionality Reduction, Clustering, and Annotation

To standardize the transcriptomic profiles across cells, we applied SCTransform (v4.3.0) for normalization and variance stabilization. The FindVariableFeatures method was employed to select the 2000 most variably expressed genes. Following data scaling, we conducted Principal Component Analysis (PCA) using the first 30 principal components. To address potential batch effects and facilitate the robust integration of multi-sample datasets, we implemented the Harmony algorithm for batch correction. Batch correction adequacy was assessed according to the Local Inverse Simpson’s Index (LISI), which improved from 1.63 (pre-correction) to 1.99 (post-correction), where a value of 2 indicates perfect batch mixing. This confirms effective batch integration without over-correction, while preservation of biological variation was preserved as evidenced by distinct cell-type clusters in UMAP and the retained specificity of canonical markers. Cluster analysis with a resolution parameter of 0.5 yielded 23 distinct subpopulations. Differential gene expression analysis between these subclusters was performed using the FindAllMarkers function, applying stringent thresholds (log2 fold change <0.25 and minimum percentage threshold of 0.25). Cell-type annotation was then carried out by cross-referencing the identified marker genes with established signatures in the CellMarker database, enabling precise cellular classification.

### 2.3. Single-Cell Function Enrichment Analysis and Metabolism Analysis

To investigate functional pathways, we conducted KEGG enrichment analysis on differentially expressed genes (DEGs) in CD8 effector memory T cells from principal study groups using the clusterProfiler software (version 4.12.6). For metabolic characterization at single-cell resolution, we employed the scMetabolism (version 0.2.1) package to compare metabolic pathway activities among three distinct T cell populations: CLSTN3-positive CD8 EM T cells, CLSTN3-negative CD8 EM T cells, and non-CD8 EM T cell subsets.

### 2.4. Cell–Cell Communication Analysis and Pseudotime Analysis

To investigate intercellular signaling networks, we employed the CellChatDB.human database and identified “Secreted Signaling” as the primary communication mechanism. We then applied the computeCommunProb algorithm to systematically calculate interaction probabilities between different cellular subtypes.

After the preprocessing steps, which included gene expression binarization and the detection of significantly expressed genes, we utilized the monocle toolkit (v2.32.0) to perform detailed pseudotemporal trajectory analysis, enabling the reconstruction of cellular developmental pathways and transitional states.

### 2.5. Two-Sample Mendelian Randomization and Colocalization Analysis

For our ovarian cancer investigation, we systematically acquired GWAS summary statistics from two key studies (GCST90436511 [25] and GCST9001821 [26]) through the GWAS catalog database (https://www.ebi.ac.uk/gwas/) (accessed on 20 October 2025). We further supplemented our analytical framework by obtaining CLSTN3 expression quantitative trait loci (eQTL) data from the OpenGWAS platform (https://opengwas.io) (accessed on 20 October 2025).

We implemented Mendelian randomization analyses utilizing the TwoSampleMR package within R software version 4.4.1. Our analytical approach incorporated five established MR methodologies—inverse variance weighted (IVW), weighted median, simple mode, weighted mode, and MR-Egger regression—with IVW designated as our principal analytical strategy. To ensure result reliability, we performed leave-one-out sensitivity analyses to identify potential influential single-nucleotide polymorphisms. Additionally, we conducted heterogeneity assessments and horizontal pleiotropy evaluations to verify result robustness. Multiple comparison correction was applied using Benjamini–Hochberg FDR and Bonferroni methods. All statistical outcomes are reported as odds ratios accompanied by 95% confidence intervals. Statistical significance was defined as *p*-values below 0.05.

### 2.6. Flow Cytometry Analysis

Peripheral blood mononuclear cells (PBMCs) were isolated from five ovarian cancer patients and five healthy controls. For intracellular cytokine staining, PBMCs were stimulated with phorbol 12-myristate 13-acetate (PMA, 50 ng/mL) and ionomycin (1 μg/mL) in the presence of brefeldin A (10 μg/mL) for 4–6 h at 37 °C with 5% CO_2_. Cells were then harvested and stained with surface antibodies (anti-CD45, anti-CD3, anti-CD8) for 30 min at 4 °C. Following fixation and permeabilization, data were analyzed using the FlowJo v10.8 software. CD8^+^ TNF-α^+^ effector T cells were gated as CD45^+^CD3^+^CD8^+^TNF-α^+^ cells, while CLSTN3^+^ CD8^+^ TNF-α^+^ effector T cells were gated as CD45^+^CD3^+^CD8^+^TNF-α^+^ CLSTN3^+^ cells.

### 2.7. Cell Culture and siRNA Transfection

The human ovarian cancer cell line HOC7 was maintained in RPMI-1640 medium supplemented with 10% fetal bovine serum (FBS), 100 U/mL penicillin, and 100 μg/mL streptomycin at 37 °C in a humidified incubator with 5% CO_2_. Cells were seeded in 6-well plates at a density of 2 × 10^5^ cells per well and transfected with three independent small interfering RNAs (siRNAs) targeting CLSTN3 (siCLSTN3-1, siCLSTN3-2, siCLSTN3-3) or a scrambled negative control (siNC) using Lipofectamine 3000 according to the manufacturer’s protocol. Cells were harvested at 72 h post-transfection for downstream analyses.

### 2.8. Quantitative Real-Time PCR (qRT-PCR)

Total RNA was extracted using RNA-easy Isolation Reagent (Vazyme, Nanjing, China) and reverse-transcribed into cDNA using the HiScript III RT SuperMix for qPCR (Vazyme, Nanjing, China). qRT-PCR was performed on a QuantStudio 7 Flex system using ChamQ Universal SYBR qPCR Master Mix (Vazyme, Nanjing, China) with gene-specific primers for CLSTN3 and GAPDH (internal control). Relative mRNA expression was calculated using the 2^−ΔΔCt^ method and normalized to the siNC group.

### 2.9. Flow Cytometry Validation of CLSTN3 Knockdown

At 72 h post-transfection, HOC7 cells were harvested and stained with anti-CLSTN3 antibody conjugated to APC. Knockdown efficiency was calculated as the percentage reduction in MFI relative to siNC.

### 2.10. Cell Proliferation Assay (CCK-8)

Cell proliferation was assessed using the Cell Counting Kit-8 (CCK-8) assay. HOC7 cells were seeded in 96-well plates at a density of 3 × 10^3^ cells per well and transfected with siCLSTN3 or siNC. At 72 h post-transfection, 10 μL of CCK-8 solution was added to each well, and cells were incubated for an additional 2 h at 37 °C. Absorbance was measured at 450 nm using a microplate reader. Cell viability at 72 h was normalized to the siNC group and is expressed as relative survival rate (%).

### 2.11. Statistical Analysis

Data are presented as mean ± standard deviation (SD) or mean ± standard error of the mean (SEM), as indicated. Comparisons between two groups were performed using the unpaired Student’s *t*-test or Mann–Whitney U test. Comparisons among multiple groups were analyzed using one-way ANOVA followed by Tukey’s post hoc test. Statistical significance was defined as *p* < 0.05. All analyses were conducted using the GraphPad Prism 9.0 or R 4.4.1. All R code used for data preprocessing, statistical analysis, and figure generation is publicly available at GitHub (https://github.com/liuchen502/MDD-OC/tree/main) (accessed on 13 May 2026).

## 3. Results

**Figure 1:**
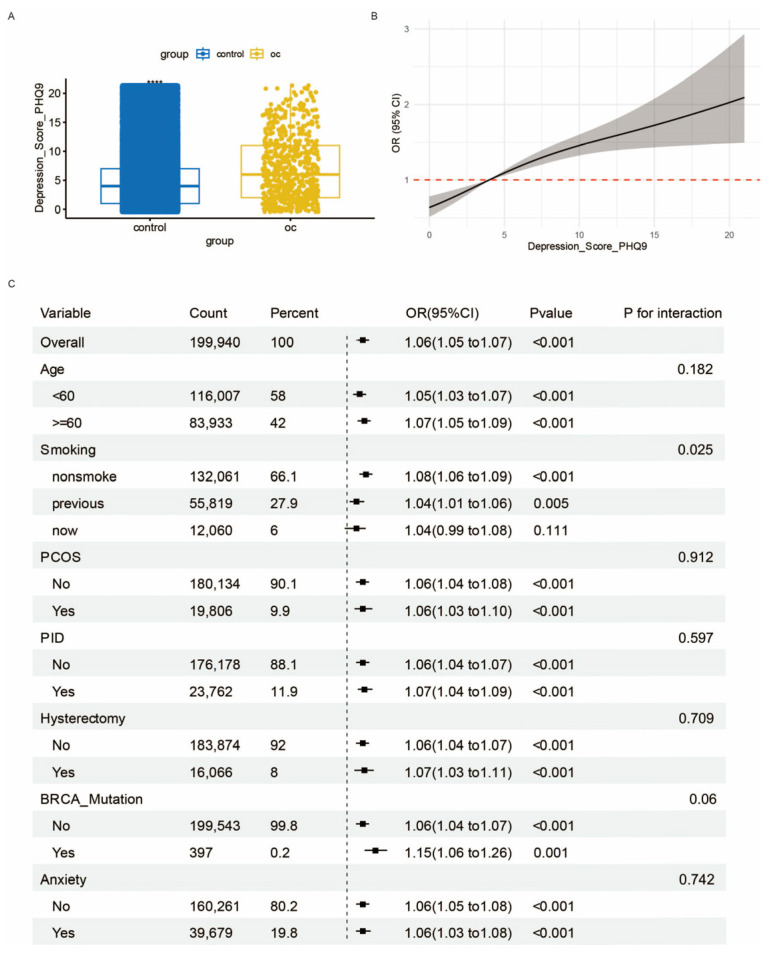
Association between depression and ovarian cancer risk in the UK Biobank cohort. (**A**) Bar graph showing depression scores in participants with ovarian cancer versus controls. Data are presented as mean ± SD. Statistical significance was determined by unpaired Mann–Whitney U test. **** *p* < 0.0001. (**B**) Restricted cubic spline (RCS) plot depicting the nonlinear relationship between depression scores and ovarian cancer risk from logistic regression. The grey shades represent the 95% confidence interval (CI). (**C**) Forest plot of subgroup analyses examining the interaction between depression and various risk factors on ovarian cancer incidence. Data are presented as odds ratios (ORs) with 95% confidence intervals (CIs).

Utilizing UK Biobank data, we investigated the association between depression and ovarian cancer among 625 ovarian cancer patients and 199,315 matched controls. PHQ-9 scores were significantly higher in the OC group than in the controls (*p* < 0.001, Figure 1A). RCS analysis revealed a positive nonlinear dose–response relationship between depression scores and OC risk (Figure 1B), where each one-unit increase in PHQ-9 score was associated with a 6% higher OC risk (OR = 1.06, 95% CI: 1.05–1.07, *p* < 0.001, Figure 1C). A significant interaction was observed for smoking status (*p* = 0.025), with the strongest effect in never-smokers (OR = 1.08, 95% CI: 1.06–1.09), and a marginally significant interaction was found for BRCA mutation status (*p* = 0.06; carriers: OR = 1.15 vs. non-carriers: OR = 1.06). No significant interactions were detected for age, PCOS, PID, hysterectomy, or anxiety (all *p* > 0.05), though the positive association remained significant across all subgroups. These findings indicate that depression is a risk factor for ovarian cancer.

**Figure 2:**
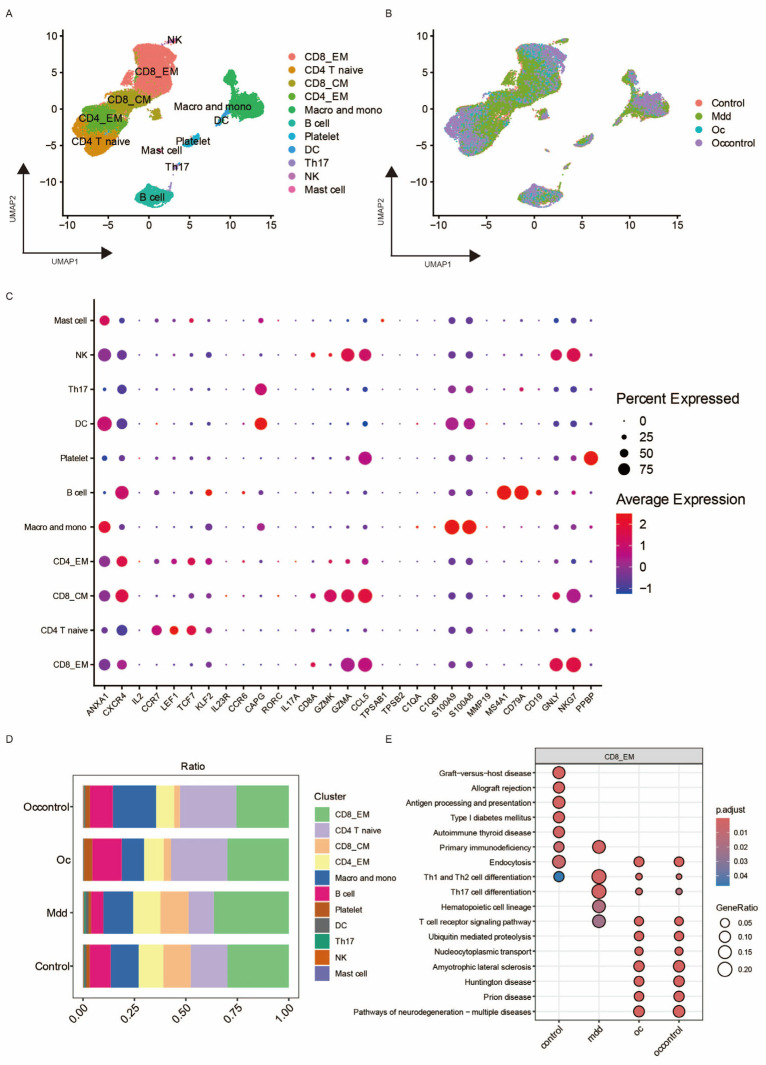
Comprehensive scRNA-seq analysis unveils the translational landscape and CD8_EM T cell heterogeneity in PBMCs of MDD and OC. (**A**) UMAP plot of single cells derived from peripheral blood samples, with cells colored and sized according to major cell types. (**B**) UMAP visualization of single cells from peripheral blood mononuclear cells (PBMCs), with cells colored and sized according to major sample groups. (**C**) Dot plot utilized for identifying marker genes across various cell subtypes. (**D**) Cell proportion diagram illustrating the distribution of 11 cell groups across control, ovarian cancer, and Major Depressive Disorder (MDD) PBMC samples. (**E**) Bubble chart displaying the KEGG pathway analysis results for CD8 Effector Memory (EM) cells across four different groups, where the bubble size corresponds to the “GeneRatio” and the color indicates the adjusted pval (*p*.adjust).

To investigate the role of peripheral immunity in major depressive disorder (MDD) and ovarian cancer (OC), we constructed a single-cell transcriptomic compendium by aggregating publicly available peripheral blood mononuclear cell (PBMC) datasets. Following rigorous quality filtering, samples from four distinct cohorts—OC patients, matched healthy controls, MDD patients, and corresponding controls—were harmonized using Harmony, effectively removing technical artifacts while retaining biologically relevant variation (Figure 2B). Unsupervised clustering delineated 11 transcriptionally discrete cell populations, with identities confirmed through established lineage markers (Figure 2A,C). Given the predominance of T-lineage cells, we conducted an in-depth characterization of their subset distribution.

Notably, CD8_EM T cells exhibited selective expansion and phenotypic convergence in both MDD and OC, a pattern absent in the control groups (Figure 2D). This unanticipated immunological parallel between a psychiatric condition and a solid tumor suggests a common immune profile that transcends conventional disease classifications. Pathway analysis within this subset uncovered a coordinated enhancement of T cell receptor (TCR) signaling in both disorders, indicating a potential mechanistic nexus connecting two clinically distinct entities (Figure 2E). Based on these observations, we focused subsequent investigations on CD8_EM T cells to elucidate the transcriptional networks and regulatory mechanisms governing their co-expansion in depression and cancer, and to assess whether shared immune mediators contribute to disease mechanisms across these disparate conditions.

**Figure 3:**
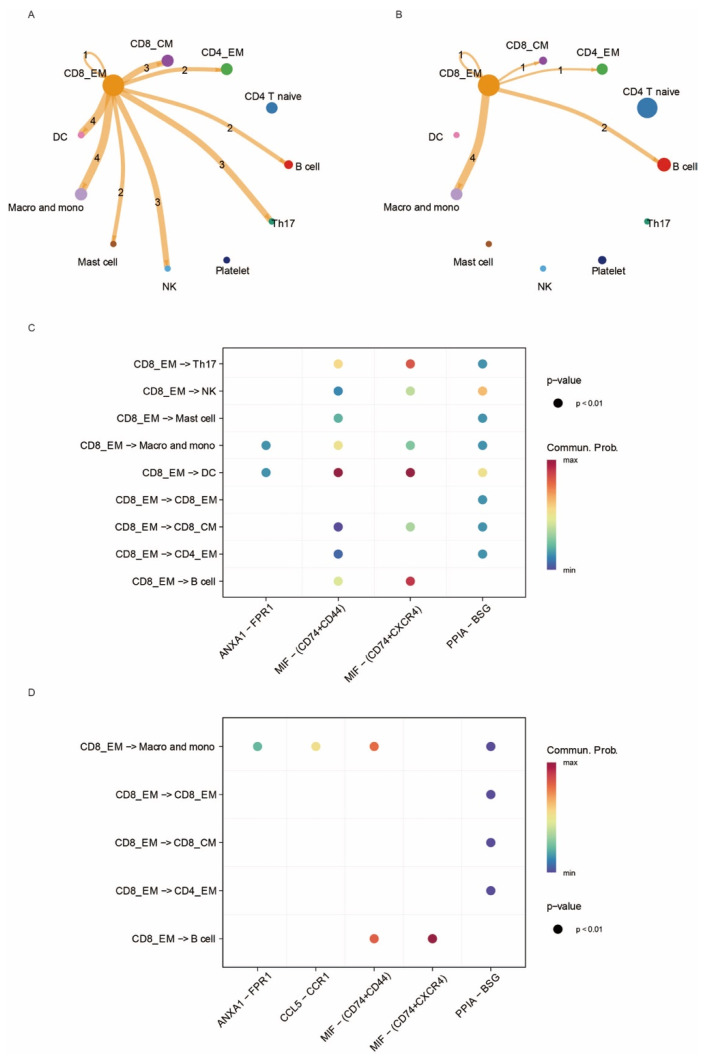
Deciphering the complex interaction of CD8 EM T cells in PBMC of ovarian cancer and MDD patient. (**A**) The CellChat network depicting interactions between CD8 EM T cells and other cell types in the PBMCs of patients with Major Depressive Disorder (MDD). The numbers on the edges represent the number of significant ligand-receptor interactions between CD8_EM T cells and each cell type (**B**) The cell–cell interaction network in the PBMCs of patients with Ovarian Cancer (OC). The numbers on the edges represent the number of significant ligand-receptor interactions between CD8_EM T cells and each cell type. (**C**) Dotplot illustrating ligand–receptor pathways involving CD8 EM T cells and other cell types in the PBMCs of patients with MDD. (**D**) Ligand–receptor pathways enriched in the PBMCs of patients with OC.

To delineate the intercellular networks associated with CD8_EM T cell expansion in OC and MDD, we constructed an integrated cellular communication map using CellChat. Despite the clinical differences between these diseases, CD8_EM T cells formed nearly identical multicellular communication hubs in both settings, engaging with CD4^+^ EM, CD8^+^ central memory (CM) T cells, B cells, monocytes, and macrophages with highly consistent interaction strengths (Figure 3A,B).

Ligand–receptor analysis further identified a conserved molecular repertoire: in both OC and MDD, CD8_EM T cells predominantly secrete MIF, which is selectively received by CD74^+^CD44^+^ monocyte–macrophage populations, while a complementary MIF–CD74^+^CXCR4^+^ axis facilitates crosstalk with B cells. Concurrently, PPIA–BSG interactions mediate communication between CD8_EM and both CD4_EM and CD8_CM T cell subsets (Figure 3C,D). The striking preservation of these MIF- and PPIA-centered networks across oncological and neuropsychiatric contexts identifies them as fundamental microenvironmental regulators that promote CD8_EM T cell accumulation and functional specialization in both disease states.

**Figure 4:**
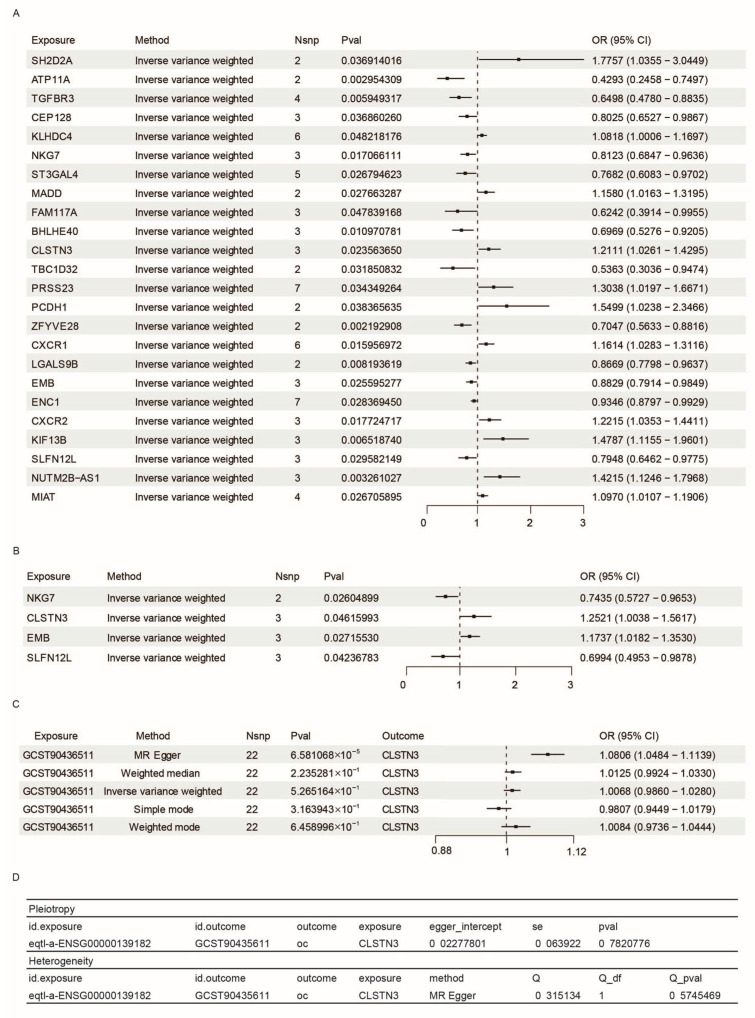
MR analysis results between CD8_EM specific genes EQTLs and ovarian cancer GWAS data. (**A**) Forest plot of a MR analysis using the Inverse Variance Weighted (IVW) method, identifying CD8 EM specific eQTL associated with the GWAS study GCST90436511, with *p* < 0.05. (**B**) MR analysis outcomes via the IVW method, highlighting CD8 EM specific genes linked to GWAS study GCST9001821 with *p* < 0.05. (**C**) Bidirectional causal relationship underlying CLSTN3 eQTL (eqtl-a-ENSG000139182) and Ovarian Cancer GWAS study GCST90436511. (**D**) Table presenting the horizontal pleiotropy and heterogeneity of CLSTN3 on GCST90436511.

**Figure 5:**
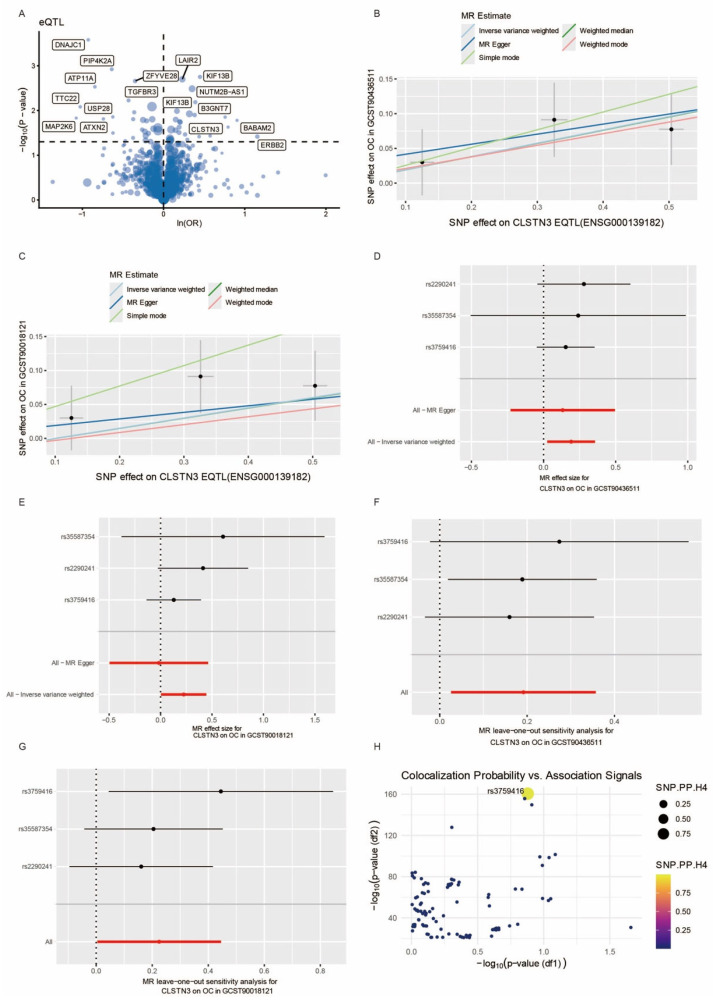
MR and coloc analysis of CLSTN3 EQTL (N = 31684) and ovarian cancer GWAS data. (**A**) Volcano plot displaying the results of a Mendelian Randomization (MR) analysis, identifying CD8 Effector Memory (EM) specific gene Expression Quantitative Trait Loci (eQTL) associated with the GWAS study GCST90436511 (sample size = 391,798). (**B**) Scatter plot illustrating the effects of CLSTN3 eQTL SNPs on the GWAS study GCST90436511, where the slope of each line corresponds to the estimated MR effect per method. (**C**) Scatter plot depicting the effects of CLSTN3 eQTL SNPs on the GWAS study GCST90018121 (sample size = 411,609). (**D**) Forest plot presenting individual and combined MR-estimated effect sizes of CLSTN3 eQTL SNPs on the GWAS study GCST90436511. (**E**) Forest plot presenting individual and combined MR-estimated effect sizes of CLSTN3 eQTL SNPs on the GWAS study GCST90018121. (**F**) Forest plot showcasing the results of a leave-one-out sensitivity analysis for CLSTN3 eQTL SNPs in an Ovarian Cancer (OC) GWAS study (GCST90436511). (**G**) Forest plot showing the results of a leave-one-out sensitivity analysis for CLSTN3 eQTL SNPs in an Ovarian Cancer (OC) GWAS study (GCST90018121). (**H**) Scatter plot visualizing the relationship between colocalization probability and association signals for genetic variants, with the x-axis representing the negative logarithm of the *p*-value for one trait and the y-axis representing the negative logarithm of the *p*-value for another trait. The point size indicates the SNP’s Posterior Probability of H4 (PP_H4), and the color represents the PP_H4 value.

To elucidate the molecular mechanisms underlying ovarian cancer development, we initially identified differentially expressed genes (DEGs) by comparing CD8_EM T cells with other T cell subpopulations and with non-T-cell populations. This comparative analysis revealed 554 genes specifically expressed in CD8_EM T cells. We subsequently utilized expression quantitative trait loci (eQTL) data for these genes as genetic instruments in a two-sample Mendelian randomization (MR) framework, analyzing their association with ovarian cancer risk using the largest available GWAS dataset (GCST90436511). Our analysis identified 24 candidate genes significantly linked to ovarian cancer susceptibility (Figure 5A), comprising 11 risk-enhancing and 13 protective variants (Figure 4A). No genes survived multiple-testing correction (FDR q < 0.05; Bonferroni *p* < 0.05) (Table S1). We therefore prioritized candidates by convergent genetic and biological evidence rather than statistical significance alone [27].

To validate these associations and mitigate potential population stratification effects, we performed replication analyses using an independent GWAS cohort (GCST90018121). Four genes demonstrated consistent effects, with CLSTN3 showing the most robust association–its eQTL variants consistently elevated ovarian cancer risk in both discovery and validation datasets (Figure 4B). Comprehensive sensitivity analyses confirmed the absence of pleiotropic effects or significant heterogeneity, supporting the validity of our causal estimates (Figure 4D).

We further investigated potential reverse causality by applying reverse MR to examine whether ovarian cancer status influences CLSTN3 expression. While MR-Egger analysis showed marginal evidence for bidirectional effects, four alternative analytical approaches failed to support reverse causation (Figure 4C), suggesting that CLSTN3 primarily functions as a risk factor for ovarian cancer rather than an ovarian cancer-mediated consequence.

The integration of MR results from both GWAS datasets (GCST90436511 and GCST90011821) demonstrated consistent positive associations between genetically predicted CLSTN3 expression and ovarian cancer risk (Figure 5B–E). Leave-one-out sensitivity analyses confirmed the stability of these associations, with effect estimates remaining significant after the iterative exclusion of individual variants (Figure 5F,G). Colocalization analysis provided additional evidence, revealing shared genetic architecture between CLSTN3 eQTL and ovarian cancer GWAS signals at rs3759416 (PPH_4_ = 99.99%, Figure 5H), indicating that this variant likely regulates both CLSTN3 expression and disease susceptibility. These findings collectively establish CLSTN3 as a promising ovarian cancer susceptibility gene whose expression is modulated by the rs3759416 variant.

**Figure 6:**
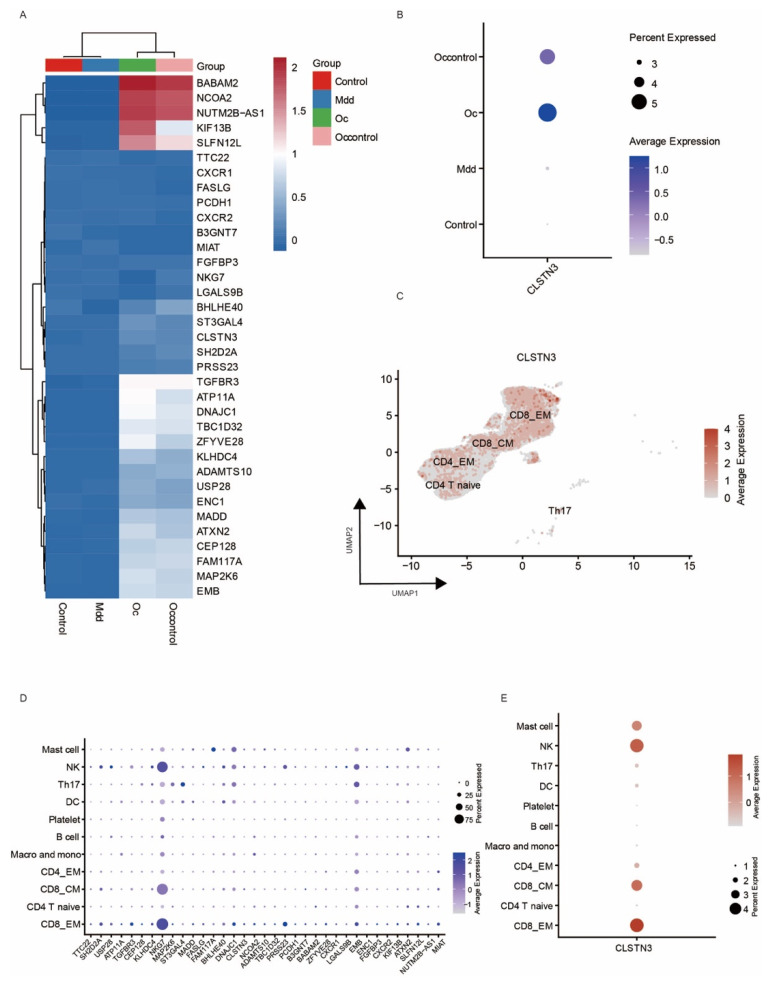
Potential gene patterns and CLSTN3 expression level. (**A**) Heatmap showing the average expression levels of select genes across different groups (Control, Mdd, and Occontrol, Oc). The color gradient from blue to red indicates expression levels from low to high. (**B**) Dot plot illustrating the average expression levels of CLSTN3 in different groups (Control, Mdd, and Occontrol, Oc). The size of the dots corresponds to the number of samples expressing the gene, and the color gradient represents the average expression level. (**C**) UMAP plot visualizing the distribution of major cell types, color-coded by the average expression levels of CLSTN3. The color gradient from grey to red indicates expression levels from low to high. (**D**) Dot plot showing the expression levels of CD8 EM specific and MR-screening positive genes across major cell types. (**E**) Dot plot representing the expression levels of CLSTN3 across major cell types.

Figure 5A presents a comprehensive heatmap analysis of differentially expressed genes associated with both MDD and ovarian cancer. Notably, CLSTN3 demonstrated significant overexpression in affected individuals compared to healthy controls (Figure 5B). Single-cell resolution analysis (Figure 6D) identified CD8_EM T cells as exhibiting the highest CLSTN3 expression levels among all examined cell populations, as visualized in the accompanying dot plot (Figure 6C,E).

**Figure 7:**
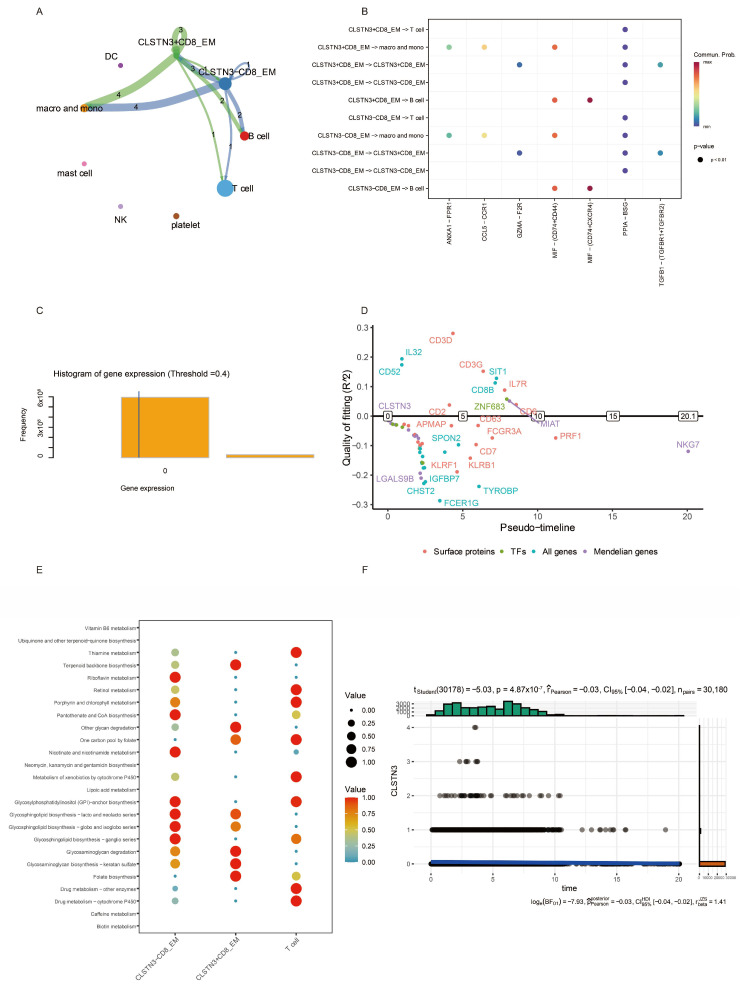
Potential physiological function of CLSTN3 gene in the comorbidity of depression and ovarian cancer disorders. (**A**) Circle network visualizing interaction between CLSTN3-negative and CLSTN3-positive CD8 EM T cells and other cell types in PBMCs. (**B**) Dotplot representing ligand–receptor mediating interaction between CLSTN3-negative and CLSTN3-positive EM T cells, and other cell types in PBMCs. (**C**) Histogram showing binarized gene expression data distribution and pseudotime analysis with a threshold of 0.4. (**D**) The expression trajectories of represented genes along the pseudotime of CD8 EM T cells. (**E**) Dotplot illustrating metabolic pathway enrichment analysis for CLSTN3-negative and CLSTN3-positive CD8 EM T cells and other T cell types. (**F**) Scatter plot depicting the relationship between CLSTN3 gene expression and pseudotime.

Subsequent CellChat and pseudotemporal trajectory analyses were performed to investigate CLSTN3’s functional involvement in ovarian cancer pathogenesis. Our results revealed comparable intercellular communication patterns between CLSTN3-positive and negative cells across most immune cell types, including macrophages/monocytes, B cells, and various T cell subsets, with the exception of CD8_EM T cells. The ligand–receptor interaction profiles showed remarkable similarity between these populations, suggesting CLSTN3’s limited impact on CD8_EM T cell-mediated immune crosstalk (Figure 7A,B).

Pseudotime trajectory reconstruction of CD8_EM T cell development identified distinct temporal expression patterns of key regulatory genes. Early developmental stages were characterized by a robust expression of LGALS9B, CD3D, FCER1G, CHST2, and IGFBP7 following expression threshold optimization (Figure 7C). While CLSTN3 demonstrated a statistically significant but biologically negligible negative correlation with pseudotime (r = −0.03), most marker genes exhibited poor trajectory fitting, indicating minimal temporal regulation (Figure 7D).

Metabolic pathway analysis uncovered significant differences between CLSTN3-expressing and non-expressing CD8_EM T cells. CLSTN3-positive cells showed enrichment in folate-related metabolic processes, glycan catabolism, and terpenoid biosynthesis, whereas their negative counterparts displayed preferential activation of vitamin-related and coenzyme A biosynthesis pathways (Figure 7F).

**Figure 8:**
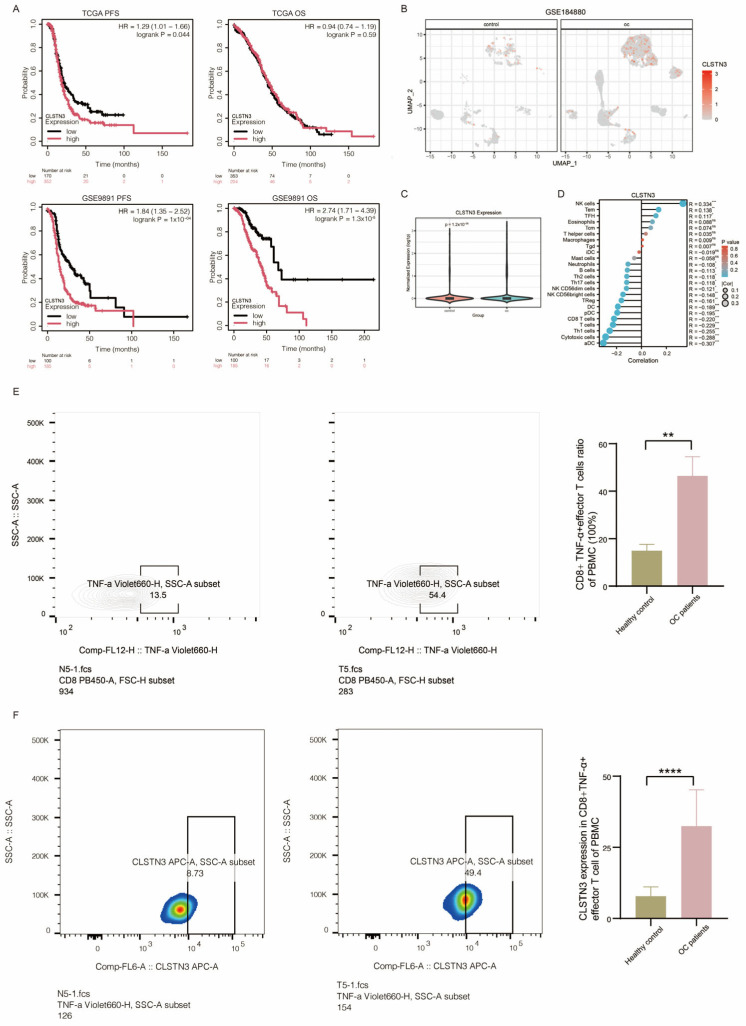
Expression of CLSTN3 and Survival in Ovarian Cancer. (**A**) Survival curves of CLSTN3 expression of oc in TCGA and GSE9891. (**B**) UMAP visualization of CLSTN3 expression in normal ovarian and ovarian cancer tissues from the GSE184880 dataset. (**C**) Violin presentation of CLSTN3 expression in normal ovarian and ovarian cancer tissues from the GSE184880 dataset. (**D**) Correlation between CLSTN3 expression and immune cell infiltration in TCGA ovarian cancer cohort. (**E**) Representative flow cytometry plots showing the gating strategy for CD8^+^ TNF-α^+^ effector T cells (CD3^+^CD8^+^CD45^+^ TNFα^+^) in peripheral blood mononuclear cells (PBMCs) from a healthy donor and an ovarian cancer patient (**Left**). Quantification of CD8^+^ TNF-α^+^ effector T cells proportions among total CD8^+^ T cells in healthy controls (n = 5) and ovarian cancer patients (n = 5). Data are presented as mean ± SD. Statistical significance was determined by unpaired Mann–Whitney U test. ** *p* < 0.01 (**Right**). (**F**) Representative flow cytometry plots showing the gating strategy for CLSTN3^+^ in CD8^+^ TNF-α^+^ effector T cells (CD3^+^CD8^+^CD45^+^TNFα^+^CLSTN3^+^) in peripheral blood mononuclear cells (PBMCs) from a healthy donor and an ovarian cancer patient (**Left**). Quantification of CLSTN3^+^proportions among total CD8^+^ EM T cell in healthy controls (n = 5) and ovarian cancer patients (n = 5). Data are presented as mean ± SD. Statistical significance was determined by unpaired Mann–Whitney U test. **** *p* < 0.0001 (**Right**).

To explore the role of CLSTN3 in ovarian cancer, we analyzed OS and PFS data from TCGA and GSE9891. CLSTN3 expression was significantly associated with worse PFS in TCGA (HR = 1.29, 95% CI: 1.01–1.66, *p* = 0.044) and with both PFS (HR = 1.84, 95% CI: 1.35–2.52, *p* = 1 × 10^−4^) and OS (HR = 2.74, 95% CI: 1.71–4.39, *p* = 1.3 × 10^−5^) in GSE9891 (Figure 8A). Single-cell analysis (GSE184880) confirmed elevated CLSTN3 in ovarian cancer tissues (Figure 8B,C), and TCGA data showed a positive correlation between CLSTN3 expression and CD8^+^ EM T-cell infiltration (R = 0.138, *p* < 0.01, Figure 8D). Flow cytometry of PBMCs from five ovarian cancer patients and five healthy controls revealed higher proportions of CD8^+^ TNF-α^+^ effector T cells in patients (*p* < 0.01), with markedly elevated CLSTN3 expression in this subset (*p* < 0.0001, Figure 8E,F), consistent with our peripheral blood scRNA-seq findings (Figure 2D).

**Figure 9:**
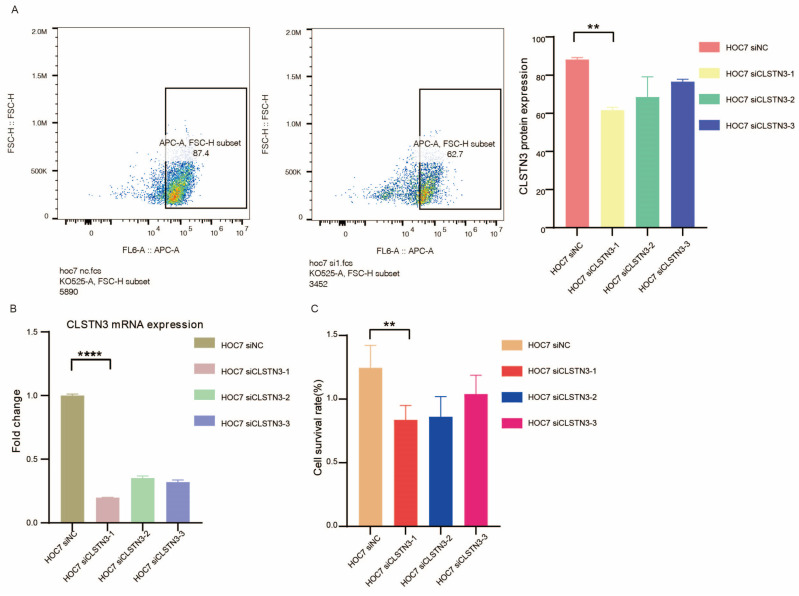
In Vitro Validation of the Effect of CLSTN3 on Ovarian Cancer Cell Proliferation. (**A**) Flow cytometry histograms showing CLSTN3 protein expression in ovarian cancer cells transfected with si-NC (negative control) and si-CLSTN3 (Left). Bar graph showing significant reduction in both siCLSTN3 groups compared to si-NC, ** *p* < 0.01 (Right). (**B**) qRT-PCR analysis of CLSTN3 mRNA expression in si-NC and si-CLSTN3 transfected cells, normalized to GAPDH. Data are presented as mean ± SD from three independent experiments. **** *p* < 0.0001. (**C**) Quantitative bar graph illustrating relative cell viability at 72 h after transfection with si-NC or si-CLSTN3. Viability was determined by CCK-8 assay and expressed as a percentage of the si-NC control. Values are mean ± SD (n = 4). Statistical significance was determined by unpaired Student’s *t*-test. ** *p* < 0.01.

In vitro, CLSTN3 knockdown in HOC7 cells using siRNA significantly attenuated proliferation (*p* < 0.01, Figure 9A–C), indicating that CLSTN3 promotes ovarian cancer cell proliferation.

## 4. Discussion

Ovarian cancer and major depressive disorder (MDD) are twin scourges that erode both individual well-being and the fabric of society, exacting an ever-rising toll through healthcare costs and lost human potential [28,29]. As illustrated in Figure 1, depression is identified as a significant risk factor for ovarian cancer, with a significant dose–response relationship. This association is particularly pronounced among never-smokers and may be amplified in BRCA mutation carriers. Contemporary epidemiological investigations have shifted the paradigm regarding MDD’s role-from passive comorbidity to an active pathogenic contributor [30]. A comprehensive multivariate study demonstrated that women with documented depressive episodes occurring 2–4 years before cancer detection exhibited a 30% elevated risk of ovarian cancer development (adjusted HR = 1.30, 95% CI 1.05–1.60) [31]. Our experimental findings substantiate these clinical observations, providing mechanistic evidence for the psychoneuroimmunological interplay between mood disorders and oncogenesis.

The pathophysiological understanding of MDD has evolved beyond neurotransmitter dysregulation to encompass systemic perturbations involving sustained β-adrenergic activation and chronic immune dysfunction [32]. Chronic psychological stress promotes ovarian tumor progression through β-adrenergic receptor-mediated cAMP-PKA pathway activation, enhancing angiogenesis and neoplastic proliferation [33]. Concurrently, emerging evidence has revealed depression’s profound impact on tumor immunology [34]. Depression-associated alterations in the tumor immune microenvironment (TIME) include cytokine polarization—the shift from IFN-γ-producing CD8^+^ T cells to IL-4-secreting subsets—creating an immunosuppressive niche favorable for malignant cell survival [35].

Our immunological profiling reveals striking parallels: circulating CD8_EM T cell populations exhibit comparable expansion patterns in both MDD and ovarian cancer patients, with quantitatively matched communication patterns among CD4_EM, CD8_CM, B lymphocytes, monocytes, and macrophages. This conserved immune signature presents a potential diagnostic biomarker and therapeutic target for the MDD–ovarian cancer comorbidity. Future investigations should focus on deciphering the precise mechanistic pathways underlying this clinical association.

Furthermore, our research reveals CLSTN3—a synaptic adhesion molecule traditionally associated with neuronal connectivity—as a novel biomarker on CD8_EM T cells that modulates ovarian cancer susceptibility. Originally characterized by its role in synaptic organization through neurexin interactions [36], CLSTN3 demonstrates broader biological significance: its DNA methylation patterns correlate with structural brain changes in MDD, suggesting potential cross-tissue epigenetic regulation [37]. Although CLSTN3 has been implicated in neurodegenerative processes through amyloid plaque formation, its potential oncogenic properties have remained unexplored.

If validated as a functional mediator, CLSTN3 could theoretically present opportunities for therapeutic intervention. As a cell surface adhesion molecule, it represents a putative target for future drug development efforts, contingent upon the resolution of selectivity, delivery, and safety challenges. The rs3759416 variant–CLSTN3 expression axis raises the possibility of genetic risk stratification, though this remains speculative and requires prospective clinical validation [38], while epigenetic strategies targeting CLSTN3 DNA methylation represent another innovative frontier [39]. However, numerous unresolved challenges would need to be addressed before any therapeutic application could be considered. The three-dimensional structure of CLSTN3’s extracellular domain remains unknown, and its binding partners in immune cells—beyond the well-characterized neurexin interactions in neurons—have not been identified. Given CLSTN3’s expression in the central nervous system, on-target off-tumor effects pose a significant safety concern. Furthermore, blood–brain barrier penetration remains a substantial hurdle for systemic drug delivery. To our knowledge, no CLSTN3-specific therapeutics have entered clinical development.

In the following, we outline three speculative directions for future investigation. First, disease-context-dependent targeting may be warranted given the divergent expression patterns of CLSTN3 in ovarian cancer versus MDD. Second, pharmacological intervention directed at CLSTN3-mediated signaling pathways in T cells represents a theoretical possibility, though the precise mechanisms remain undefined. Third, RNA-targeted approaches such as siRNA or antisense oligonucleotides might enable cell type-specific modulation, contingent upon solving delivery specificity for CD8_EM T cells. Major barriers include neuronal expression with attendant CNS side-effect risk and potential functional redundancy with CLSTN1 and CLSTN2.

Several limitations of our study should be acknowledged. First, as our mechanistic findings are based on in vitro experiments using the HOC7 cell line, the absence of in vivo models precludes definitive conclusions. Notably, while CLSTN3 is highly expressed in CD8_EM T cells, functional validation was performed in HOC7 cells due to technical challenges in primary T-cell manipulation; future studies should validate its immune-regulatory function directly in CD8_EM T cells. Second, our single-cell transcriptomic analysis was limited by small sample sizes, restricting its statistical power and generalizability. Third, CLSTN3 did not withstand stringent multiple-testing correction (FDR q = 0.492), and its prioritization was driven by convergent evidence across independent GWAS datasets, strong colocalization (PPH_4_ = 99.99%), and cell-type-specific expression. This integrative approach aligns with an emerging consensus that MR-based gene discovery should incorporate the biological context beyond strict *p*-value thresholds. Fourth, while our UK Biobank analysis establishes an epidemiological association, causal directionality remains uncertain. Additionally, the CLSTN3–depression link is based on genetic association and comorbidity patterns, not direct biological validation; future bidirectional Mendelian randomization may help to clarify this. Lastly, due to the limited availability of reliable CD45RA/CCR7 antibodies for our panel, CD8^+^ effector memory T cells were functionally identified as CD3^+^CD8^+^TNF-α^+^ cells following PMA/ionomycin stimulation, rather than by the canonical surface markers. This approach captures activated/effector CD8^+^ T cells with cytokine-producing capacity, rather than the strictly defined CD45RA^−^CCR7^−^ subset.

Translational Perspectives: Future research should prioritize prospective longitudinal designs with larger PBMC cohorts, integrate multi-omics profiling, and incorporate in vivo functional studies to unravel the precise mechanistic pathways underlying the depression–ovarian cancer axis and evaluate the therapeutic potential of targeting CLSTN3 in this comorbidity.

## Data Availability

We confirm that all summary statistics are publicly accessible via the GWAS Catalog and IEU OpenGWAS database, with exact accession numbers (Table S2), download dates, and preprocessing parameters (allele harmonization, palindromic SNP handling, genome build) provided for full reproducibility. In addition, the R code used for data processing, statistical analysis, and figure generation in this study is publicly available at GitHub: https://github.com/liuchen502/MDD-OC/tree/main (accessed on 13 May 2026).

## Supplementary Materials

The following supporting information can be downloaded at: https://www.mdpi.com/article/10.3390/biomedicines14051167/s1. Table S1. Multiple-testing correction (FDR q < 0.05; Bonferroni *p* < 0.05) of our MR analysis identified 24 candidate genes. Table S2. Summary statistics are publicly accessible via the GWAS Catalog and IEU OpenGWAS database, with exact accession numbers provided.
